# Factors contributing to moral distress among intensive care nurses: A scoping review

**DOI:** 10.12688/f1000research.127120.2

**Published:** 2024-07-15

**Authors:** Amina Mussa Ahmad, Wegdan Bani-Issa, Fatma Refaat

**Affiliations:** 1 University of Sharjah, Sharjah, United Arab Emirates

**Keywords:** End of life care, ICU, Moral distress, Powerlessness, Nurses, Teamwork, Scoping review

## Abstract

**Background:** The intensive care unit (ICU) is a busy and complex workplace, and several work-related and personal factors are known to make ICU nurses more vulnerable to moral distress than other healthcare professionals. It is crucial to identify these factors to guide future studies and preventive strategies. This scoping review explores such factors to present current knowledge on the factors that trigger moral distress and to guide future research by reviewing studies to explore and summarize factors that trigger moral distress in ICU nurses.

**Methods:** The PubMed, EBSCO, and CINAHL Plus databases were searched to identify potentially relevant studies published between 2011 to 2022. Inclusion criteria: peer-reviewed studies published in English that provided results regarding factors causes or correlated to moral distress in ICU nurses. After removing 618 duplicates, 316 papers were excluded after title and abstract screening, leaving 71 articles for full-text screening. A further 54 articles were excluded as their outcomes did not include factors that caused moral distress, or were not specific to ICU nurses, so 17 studies were eventually analysed using qualitative content analysis through an inductive approach. The findings of the articles were extracted and coded independently by two authors, and data were grouped and categorized.

**Results:** The content categories of factors contributing to ICU nurses' moral distress were organized into themes and subthemes. Four major themes were identified: Powerlessness, end-of-life care, ineffective teamwork, and personal characteristics of ICU nurses.

**Conclusions:** This review highlights the factors that contribute to moral distress in critical care nurses, which are mainly attributable to the organizational climate and the nature of the ICU clinical environment. Descriptive and intervention studies (experimental or action research) must investigate causality between identified variables to inform management strategies to improve support for ICU nurses’ coping relative to moral distress.

## Introduction

The intensive care unit (ICU) is a complex, unpredictable, and inherently stressful environment in which patient health status, clinical situations, and basic dynamics of the course of care can all change rapidly ( Johnson-Coyle *et al.,* 2016). Factors such as nurse shortages (i.e., low nurse-to-patient ratios), patient populations with high acuity, significant technological advancement, inexperienced nurses with insufficient training, and poor healthcare systems make ICU nurses particularly vulnerable to moral distress in the discharge of their duties ( Wenwen *et al.,* 2018). Moral distress is experienced frequently by all healthcare professionals, including nurses ( Chiafery *et al.,* 2018; Wenwen *et al.,* 2018). It is a phenomenon experienced when a healthcare professional knows the ethically appropriate course of action, but finds themselves unable to pursue that action, due to internal or other external constraints ( Hamric *et al.,* 2012). It occurs when conflict exits between knowledge of the ethically right course of action and institutional, departmental, interpersonal, or legal constraints, and the ability of healthcare providers to change the situation or take a course of action ( Vincent *et al.,* 2020). In particular, moral distress is linked with alleged violations of the core values and duties of the individual healthcare professional ( Colville *et al.,* 2019).

In ICUs it is typically difficult to predict the survival chances of critically ill patients, and nurses caring for such patients face far more morally distressing situations than those caring for patients with more stable conditions in other departments. ICU nurses face the twin demands of highly critical and complex biomedical service deliver alongside end-of-life issues such as sudden death, and they face moral dilemmas relating to aggressive and/or futile end-of-life interventions ( Wenwen *et al.,* 2018).

In such work environments, nurses run the risk of being involved in decisions that might be considered morally wrong ( Babamohamadi *et al.,* 2021), such as invasive interventions on patients with terminal illnesses, requesting pointless tests or examinations, inadequate and ineffective treatment by colleagues, a lack of organizational support, and absence of balance of power among healthcare professionals, all of which have been identified as causative factors in moral distress among nurses ( Berhie *et al.,* 2020; Emple *et al.,* 2021; Heydari *et al.,* 2018; Robaee *et al.,* 2018). Other factors include giving life-supporting treatments that prolong the process of dying, and adhering to relatives’ directions to proceed with life-supporting medicines that may not be in the best interests of patients themselves ( Wenwen *et al.,* 2018). All of these factors lead to inability of healthcare team to reach consensus regarding to the plan of care for patients, resulting in team conflict and morally distressing situations ( Vincent *et al.,* 2020).

Furthermore, the unpredictable progress of diseases and the fear of infection to self and family members are additional factors contributing to moral distress among ICU nurses, which was starkly revealed during the COVID-19 pandemic ( Romero-García *et al.,* 2022). In such demanding environments, burnout and low morale affects ICU nurses’ mental and physical well-being, as well as their relationships with friends and family, and patient care ( Witter *et al.,* 2020).

The consequences of moral distress in ICU nurses can include sensations of rage, dissatisfaction, powerlessness, and guilt, which are associated with discouragement, burnout, anxiety, depression, deteriorating morale and teamwork, diminishing quality of care (QoC), and difficulties associated with patient safety, all of which relate to emotional and psychological reactions correlated with moral distress ( Romero-García *et al.,* 2022). Negative personal symptoms reported by ICU nurses include nightmares, insomnia, palpitations, and neck pain ( Bani Issa *et al.,* 2021; Soleimani *et al.,* 2019). If nurses’ moral distress remains unresolved, they may become emotionally exhausted, which consecutively reduces the QoC they deliver to patients, and increases their intension to leave the profession ( McAndrew *et al.,* 2018).

As a significant issue in healthcare environments, moral distress requires prompt and rapid actions because of the danger it poses to ICU nurses’ capacity to deliver competent and ethical care, the wellbeing of service users, and the QoC provided to patients ( Almutairi *et al.,* 2019), so healthcare organizations should recognize related factors and devise solutions to prevent and resolve moral distress ( Saleh *et al.,* 2019). Nurse mangers, administrators, and policy makers should implement evidence-based strategies to lower and eliminate nurse moral distress. A high-priority recommendation is for nursing leadership to provide rigorous supports such as structured debriefings, as well as a supportive environment for counselling services and staff education, with an emphasis on addressing moral distress and coping strategies ( Dacar *et al.,* 2019). Several interventional studies aimed at reducing moral distress provided insufficient evidence on the effectiveness of related interventions to reduce the level or frequency of moral distress ( Dacar *et al.,* 2019).

Decades of research have shown that moral distress is high; however, there is little evidence of effective interventions ( Altaker *et al.,* 2018). Gaining insight into the moral distress that nurses face is the first step toward deciding actions that can provide guidance and nurse retention, which is fundamentally important for the sustainability of healthcare systems ( Karakachian and Colbert, 2019). A preliminary search of various databases such as PubMed, EBSCO, MEDLINE, and CINAHL in addition to textbooks and the Cochrane Library, revealed that several studies have been conducted to identify factors that might contribute to moral distress in healthcare professionals working in ICUs, but relatively few have explored the factors that cause moral distress among ICU nurses *per se.*

Given the impact of moral distress among ICU nurses, patient safety, and QoC ( Hou *et al.,* 2021; Wenwen *et al.,* 2018), it is crucial first to identify the factors that contribute to ICU nurses’ moral distress to guide healthcare organizations in developing resilience strategies to help ICU nurses cope with moral distress, and to pave the way for future research on practical interventions to address risk factors. Therefore, the aim of this scoping review is to present current knowledge on the factors that trigger moral distress and to guide future research by reviewing related studies to explore and summarize various factors that trigger moral distress in ICU nurses. The specific research questions that this scoping review seeks to address are:

1. What are the general characteristics of studies on factors contributing or correlated to moral distress among ICU nurses?
2. What are the factors contributing to moral distress among ICU nurses?

## Methods

### Scoping review methods

This scoping review presents the direction of existing and future studies by analysing the characteristics and contents of related research using the methodological framework of Arksey and O’Malley (2005) to review the factors that lead to moral distress. This method was chosen as a way for mapping the literature in this area, examining the breadth, scope, and nature of research activity, and identifying gaps in existing knowledge ( Harris *et al.,* 2015). The Preferred Reporting Items for Systematic Reviews and Meta-Analyses Extension for Scoping Reviews (PRISMA-ScR) checklist was used to report the present study ( Ahmad *et al.,* 2022).

### Search methods

A comprehensive search was initiated in July 2022 in the academic research databases PubMed, EBSCO host, and CINAHL Plus to review articles published in the area under study. A recent systematic review on moral distress among healthcare professionals reported a steady increase in the volume of publications on the topic since 2010 ( Lamiani *et al.,* 2017). Therefore, the search in this study scope was limited to articles published in the period between January 2010 to July 2022, in order to be able to review the most recent and relevant available scientific evidence published in this field, and to have a comprehensive understanding of the area under study and identify gaps in recent literature.

Data were collected using the main keywords on moral distress, intensive care, and nurses.

### Eligibility criteria

The inclusion and exclusion criteria are presented in Table 1. The inclusion criteria restricted the scope to peer-reviewed studies that provided results regarding factors identified as being causative in or correlated with moral distress among ICU nurses, published in English. Studies that identified factors causing general psychological distress, collection of the constructs from target groups other than ICU nurses, studies on moral distress that did not include factors causing moral distress as outcome variables, and articles published in languages other than English were excluded.

**Table 1.** Eligibility criteria as per PCC.

Eligibility Criteria Following PCC	
Inclusion	Participants Intensive care nurses Concept Factors that cause or contribute to moral distress Context 1- Studies carried out in intensive care units 2- Adult, paediatric, and neonate patients 3- Peer-reviewed articles 4- Published in English language
Exclusion	Participants Healthcare professional other than ICU nurses. Concept 1- Studies on moral distress that does not include the factors that causes moral distress as outcome variable. 2- Studies that identify factors causes general psychological distress. Context 1- Studies carried out in clinical settings other than intensive care units.

This scoping review considered both experimental and quasi-experimental study designs, along with analytical observational, descriptive observational, descriptive cross-sectional, and qualitative studies, focused on qualitative data including (but not limited to) designs such as phenomenology, grounded theory, ethnography, and qualitative description. In addition, systematic reviews that met the inclusion criteria were also considered.

### Study selection

The search results were imported into EndNote X9 and were de-duplicated based on their titles, and the automatically identified duplicates were additionally double-checked manually. Two reviewers screened the results independently against the eligibility criteria. Discrepancies were resolved through discussions or asking a third reviewer.

Two reviewers also investigated the full texts of relevant search results against the same criteria (above) involving a third reviewer in cases of disagreement. At the full-text screening stage, the reference lists of the relevant studies were also analysed to identify potential additional relevant studies not gleaned from the database searching process. Since one study may be associated with multiple reports or publications, we kept a record and cited all the reports of a single study to provide a better overview of the new research evidence.

### Data analysis

A data extraction form was created prior to data extraction to aid with the process ( Ahmad *et al.,* 2022). The form included data about the year of publication, the objectives, the country, the study design, the author(s), the participants, the sample size, the tool used in the study, and the study's findings. They were then divided into two categories for analysis, as follows: (1) general characteristics of the studies; and (2) outcome variables and findings, which demonstrated the main factors that contribute to moral distress among ICU nurses.

The general characteristics of the studies included country, measurement instrument, and study design, and results of the studies on factors contributing to moral distress were summarized, and the extracted data were documented with Microsoft Excel Version 2209 ( Table 2).

**Table 2.** Summary review of included studies.

Sample size	Country	Study design	Tool
**1-** **Prompahakul *et al.* (2021)**			
462	Thailand	Mixed-method	MMD-HP and interview
**Main findings**			
(1) Powerlessness (at patients/family, team, and organizational-levels); (2) end-of-life issues, aggressive medical interventions at the terminal stage of life, futile actions, providing false hope to patients and families, unnecessary medical treatments; (3) poor team function			
**2-** **McAndrew *et al.* (2018)**			
12 qualitative, 24 quantitative, and 6 mixed-methods studies	USA	Mixed-method review	Review paper
**Main findings**			
(1) Assisting incompetent physicians; (2) poor nurse/physician communication causing medication error; (3) unprofessional behaviour of physicians; (4) powerlessness in organizational context; (5) nursing autonomy; (6) organizational challenges; (7) communication problems; (8) moral decision-making and advocacy			
**3-** **Hiler *et al.* (2018)**			
328	USA	Descriptive correlational	MDS-R
**Main findings**			
(1) Work environment; (2) futile care; (3) powerlessness at family level; (4) nurses’ personal characteristics			
**4-** **Altaker *et al.* (2018)**			
238	USA	Descriptive correlational	Moral Distress Scale-Revised, Psychological Empowerment Index, Hospital Ethical Climate Survey, Palliative Care Delivery Questionnaire
**Main findings**			
(1) Lack of psychological empowerment; (2) negative ethical climate; (3) access to full palliative care team; (4) units size with more ICU beds			
**5- Morley *et al.*, 2022**			
21	UK	Qualitative	In-depth interview
**Main findings**			
(1) Decision making hierarchy; (2) false reassurance; (3) ethical climate; (4) conflict between professional and personal responsibilities; (5) ability to advocate; (6) team dynamics			
**6-** **Browning (2013)**			
277	Various (nurses on AACN’s e-mail newsletter list)	Cross-sectional descriptive survey design	Moral Distress Scale, Psychological Empowerment Instrument
**Main findings**			
(1) Nurses’ personal characteristics; (2) assisting incompetent physicians; (3) staff shortage; (4) aggressive treatment at end of life; (5) powerlessness at family level; (6) unnecessary medical treatment and tests; (7) false information to patient/family; (8) empowerment			
**7- Andersson *et al.* (2022)**			
71	Swedish adult ICUs	Cross-sectional	Moral Distress Scale-Revised
**Main findings**			
(1) Futile care; (2) assisting physician or nurse who provided incompetent care; (3) ethical climate; (4) deceptive communication (false hope)			
**8- Shoorideh *et al.* (2012)**			
31	Iran	Qualitative	Semi-structured, in-depth interviews of purposive sample
**Main findings**			
(1) Legal situations; (2) medical supervision (contradicting orders related to patient care among physicians); (3) accountability; (4) communication problems; (5) futile action, malpractice, and medical/care errors; (6) inappropriate responsibilities, resources, and competencies			
**9-** **Choe *et al.* (2015)**			
14	South Korea	Qualitative	In-depth face-to-face interviews
**Main findings**			
(1) Ignoring patient autonomy; (2) unnecessary medical treatments; (3) compulsory application of restraints; (4) lack of ethical sensitivity; (5)nurses’ limited autonomy; (6) conflicts with physicians; (7) conflicts with institutional policy			
**10-** **Sannino *et al.* (2019)**			
136	Italy	Cross-sectional questionnaire survey	Moral Distress Scale Neonatal-Paediatric Version
**Main findings**			
(1) Incompetent healthcare providers; (2) aggressive medical interventions at the terminal stage of life; (4) nurses’ personal characteristics; (5) number of deaths; (6) nurse with children report higher moral distress			
**11- Karanikola *et al.* (2014)**			
566	Italy	Cross-sectional correlational design	Corley’s Moral Distress Scale (CMDS)
**Main findings**			
(1) Nurse-physician collaboration and dissatisfaction on care decisions; (2) intention to resign (the frequency of occurrence of moral distress was associated with the intention of nurses to resign)			
**12-** **Asayesh *et al.* (2018)**			
117	Iran	Cross-sectional with descriptive-correlation method	Futile Care Perception Questionnaire, Corley’s Moral Distress Scale
**Main findings**			
(1) Futile care; (2) length of work experience (positive correlation)			
**13- Shoorideh & Ashktorab (2015)**			
159	Iran	Descriptive-correlation research	ICU Nurses’ Moral Distress Scale, Copenhagen Burnout Inventory, Hinshaw and Atwood Turnover Scale
**Main findings**			
Positive correlation with (1) nurses’ age; (2) work experience; (3) ratio to number of intensive care unit beds			
**14-** **Forozeiya *et al.* (2019)**			
7	Canada	Qualitative	Semi-structured interviews
**Main findings**			
(1) Low experience; (2) moral violations; (3) planning for end-of-life care; (4) withholding information from patients and families			
**15- Karagozoglu *et al.*, 2017**			
200	Turkey	Descriptive and cross-sectional	MDS-R
Main findings			
(1) Inadequate communication within the team; (2) working with professionals they considered as incompetent; (3) futile care			
**16-** **Henrich *et al.* (2016)**			
23 ICU nurses	Canada	Qualitative	Focus groups
**Main findings**			
(1) Incompetent care from other staff and physicians; (2) aggressive medical interventions; (3) contradictory care plans; (4) deciding on the end of life; (5) interactions and conflicts between staff and family; (6) lack of support from management; (7) lack of resources; (8) poor communication			
**17-** **Sannino *et al.* (2015)**			
472	Italy	Cross-sectional	Moral Distress Scale Neonatal-Pediatric Version
**Main findings**			
(1) Aggressive medical interventions for ventilator -dependent children			

Two reviewers read the included articles independently and repeatedly to gain an overall understanding of the factors contributing to moral distress. Each reviewer then independently constructed codes from the surface meaning of the text and collated it. The reviewers identified codes and themes from raw data using an inductive approach to organize the data, to reach consensus, a third reviewer resolved disagreements.

## Results

### General characteristics of the included studies

**Figure 1.**
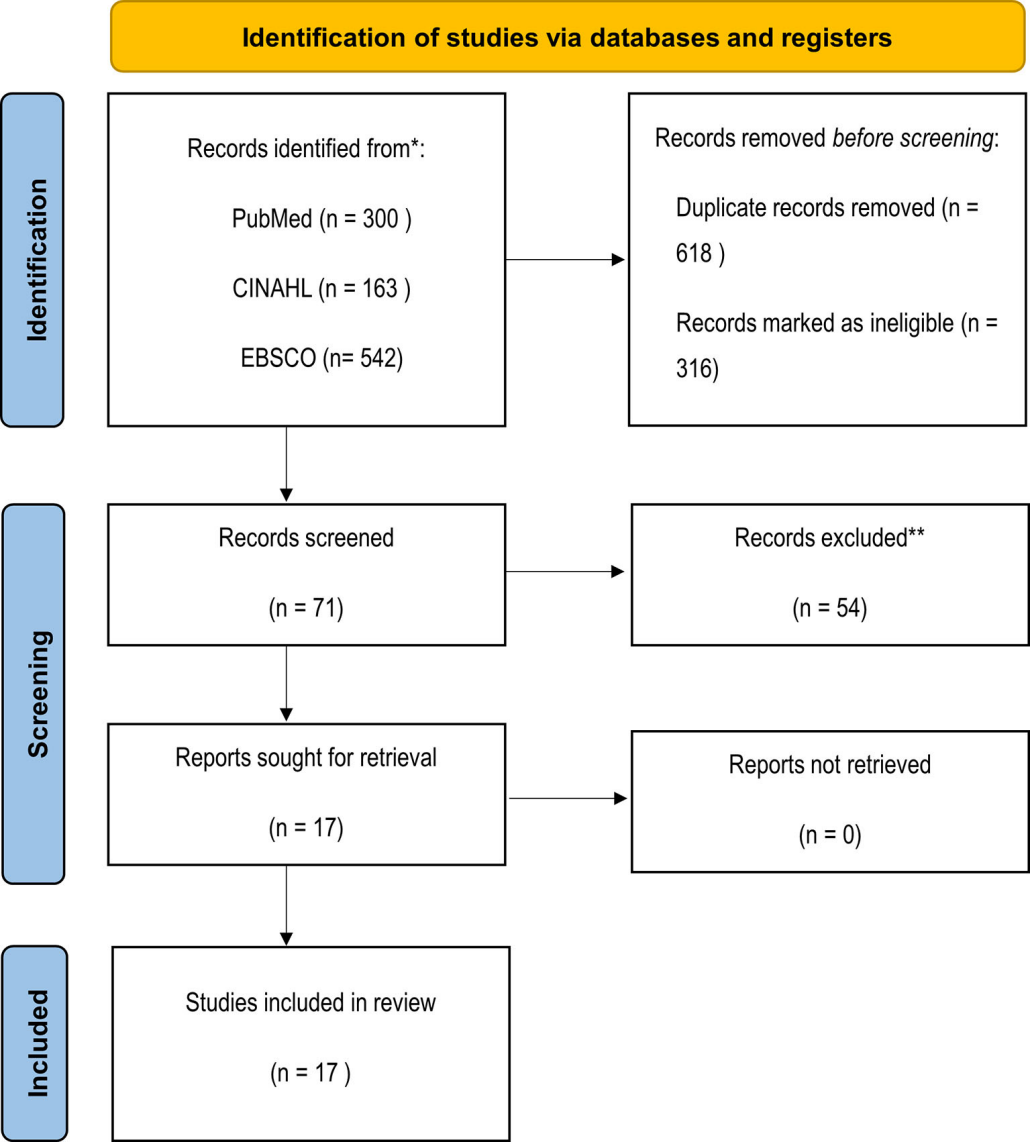
PRISMA 2020 flow diagram for new systematic reviews in databases and registers.

The initial search resulted in a total of 1,005 articles. After checking their titles and abstracts, 117 were excluded because the title of the study did not mention moral distress, 152 were excluded after reading the title (due to identifying non-eligibility factors), and 47 articles did not meet the eligibility criteria after reading their abstracts. Additionally, 618 articles were duplicates (and were removed), thus the remaining 71 articles were reviewed in full-text versions. This resulted in the exclusion of 54 articles due to their outcomes not including factors that caused moral distress or considering outcomes not specific to ICU nurses (e.g., those concerned with healthcare professionals in general or other specialties), thus 17 studies were eventually analysed (see Figure 1).

Of the 17 studies, four were conducted in the US (23.5%), three in Italy (17.6%), three in Iran (17.6%), two in Canada (11.8%), one each in South Korea (5.9%), Sweden (5.9%), Thailand (5.9%), Turkey (5.9%), and the UK (5.9%), and one was a review study ( Ahmad *et al.,* 2022).

Regarding study design, 10 studies used a descriptive correlational design (58.8%), five used a qualitative design (29%), one used mixed-methods (5.8%), and one used an analytical review design (5.8%). Finally, regarding the measurement tools used to identify factors causing moral distress in critical care nurses, five quantitative studies used the 21-item Moral Distress Scale-Revised (29.4%), two used the 21-item Moral Distress Scale Neonatal-Pediatric Version (11.8%), two used the 32-item Moral Distress Scale (11.8%), and another study used the Jameton’s Moral Distress Questionnaire (5.8%). The mixed-methods design study used the Measure of Moral Distress for Healthcare Professionals (MMD-HP) (5.8%), whereas qualitative outcomes used semi-structured interviews in five studies (29.4%) and focus groups in one study (5.8%) ( Table 3).

**Table 3.** General characteristics of the included studies.

Characteristics	n	%
Measurement Instrument		
21-item Moral Distress Scale-Revised	5	29.4%
21-item Moral Distress Scale Neonatal-Paediatric Version	2	11.8%
32-item Moral Distress Scale	2	11.8%
Jameton’s Moral Distress Questionnaire	1	5.8%
MMD-HP	1	5.8%
Semi-structured interviews	5	29.4%
Focus groups	1	5.8%
Analytical review	1	5.8%
Country of the Study		
Canada	2	11.8%
Iran	3	17.6%
Italy	3	17.6%
South Korea	1	5.9%
Sweden	1	5.9%
Thailand	1	5.9%
Turkey	1	5.9%
UK	1	5.9%
USA	4	23.5%
Design		
Descriptive correlational design	10	58.8%
Qualitative design	5	29.4%
Mixed-method	1	5.8%
Analytical review design	1	5.8%

### Factors contributing to moral distress among ICU nurses

Two reviewers grouped and categorized data from the primary independent coding of the articles; data were derived and grouped; disagreements were resolved through discussion in online meetings between the reviewers by the third reviewer; and the groups were organized into themes and subthemes. Four major themes of factors associated with moral distress in ICU nurses were identified: powerlessness, end-of-life care, ineffective teamwork, and personal characteristics of ICU nurses ( Table 4).

**Table 4.** Thematic analysis.

**Main Themes**
1- Powerlessness
**Sub-Themes**
Strong hierarchical relationship ( McAndrew *et al.*, 2018; Morley *et al.*, 2022; Prompahakul *et al.*, 2021)
Lack of empowerment ( Altaker *et al.*, 2018; Browning, 2013; Henrich *et al.*, 2016; McAndrew *et al.*, 2018; Morley *et al.*, 2022)
Negative ethical climate ( Altaker *et al.*, 2018; Andersson *et al.*, 2022; Choe *et al.*, 2015; Forozeiya *et al.*, 2019; McAndrew *et al.*, 2018; Morley *et al.*, 2022; Prompahakul *et al.*, 2021; Shoorideh *et al.*, 2012)
Poor staffing ratio ( Altaker *et al.*, 2018; Browning, 2013; Shoorideh *et al.*, 2012; Shoorideh & Ashktorab, 2015)
Nurse autonomy ( Choe *et al.*, 2015; McAndrew *et al.*, 2018; Prompahakul *et al.*, 2021)
Treatment plan directed by patient family ( Browning, 2013; Henrich *et al.*, 2016; Hiler *et al.*, 2018; McAndrew *et al.*, 2018; Prompahakul *et al.*, 2021)
2- End of life Care
**Sub-Themes**
Aggressive interventions at the terminal stage of life ( Browning, 2013; Henrich *et al.*, 2016; Prompahakul *et al.*, 2021; Sannino *et al.*, 2015, 2019)
Futile actions ( Andersson *et al.*, 2022; Asayesh *et al.*, 2018; Choe *et al.*, 2015; Hiler *et al.*, 2018; Karagozoglu *et al.*, 2017; Prompahakul *et al.*, 2021; Shoorideh *et al.*, 2012)
Providing false hope and information ( Andersson *et al.*, 2022; Browning, 2013; Choe *et al.*, 2015; Forozeiya *et al.*, 2019; Morley *et al.*, 2022; Prompahakul *et al.*, 2021)
Planning end of life care ( Forozeiya *et al.*, 2019; Henrich *et al.*, 2016)
3- Ineffective Team Function
**Sub-Themes**
Poor communication and collaboration ( Choe *et al.*, 2015; Henrich *et al.*, 2016; Karagozoglu *et al.*, 2017; Karanikola *et al.*, 2014; McAndrew *et al.*, 2018; Prompahakul *et al.*, 2021; Shoorideh *et al.*, 2012)
Incompetent healthcare providers ( Andersson *et al.*, 2022; Browning, 2013; Henrich *et al.*, 2016; Karagozoglu *et al.*, 2017; McAndrew *et al.*, 2018; Prompahakul *et al.*, 2021; Sannino *et al.*, 2019)
Contradictory orders related to patient care among physicians ( Henrich *et al.*, 2016; Shoorideh *et al.*, 2012)
Inappropriate behaviour of colleagues ( McAndrew *et al.*, 2018; Morley *et al.*, 2022; Prompahakul *et al.*, 2021)
4- Personal Characteristics
( Altaker *et al.*, 2018; Asayesh *et al.*, 2018; Browning, 2013; Forozeiya *et al.*, 2019; Hiler *et al.*, 2018; Sannino *et al.*, 2015, 2019; Shoorideh & Ashktorab, 2015)

## Discussion

The intensive care unit is a chaotic and stressful setting in which nurses are constantly confronted with disruptive factors that expose them to moral distress. This is an overwhelming environment of increasing daily admissions, transfer, and deaths; these particular stressors exacerbate underlying weaknesses common in healthcare systems worldwide, such as low nurse-patient ratios, overwork, burnout, and poor life-work balance etc., all of which are independently related with an increase in the absolute value of the moral distress of ICU nurses ( Sannino *et al.,* 2019).

From the results of the present scoping review, it is clear that there are several factors that can lead to moral distress in critical care nurses, all of which negatively affect their personal and professional lives, and consequently the QoC they deliver, resulting in poorer patient outcomes (including clinical prognosis and wellbeing), service user satisfaction, and health system efficiency.

### Powerlessness

Almost all of the articles in this review indicated that the powerlessness of ICU nurses in the face of organizational obstacles and constraints is one of the main causes of their moral distress while working with patients ( Altaker *et al.,* 2018; Andersson *et al.,* 2022; Browning, 2013; Choe *et al.,* 2015; Epstein *et al.,* 2019; McAndrew *et al.,* 2018; Morley *et al.*, 2022; Prompahakul *et al.,* 2021; Sannino *et al.,* 2019; Shoorideh *et al.,* 2012).

The most important factor contributing to differences in levels of moral stress was the prevailing ethical climate, with the creation of a positive ethical climate in the organization being associated with lower levels of moral stress ( Altaker *et al.,* 2018). ICU nurses described discomfort when work-related tasks interfered with their ability to advocate for patients (i.e., to provide holistic nursing care), or when the economic benefit of the hospital was seen as taking precedence over human life, and in these contexts the ethical climate in the organization correlated negatively with moral distress ( McAndrew *et al.,* 2018). Similarly, Choe *et al.,* (2015) revealed that administrative actions deemed to be ethically flawed often caused moral distress among participants, especially when hospitals’ financial objectives took precedence over respect for human life and rights (e.g., when patients were forced to be discharged or transferred regardless of their condition).

Related to their perceived powerlessness ( Prompahakul *et al.,* 2021), emphasized that nurses experienced moral distress within strong hierarchical organizations, wherein they lacked organizational support, which is one of the most important indicators of nursing work satisfaction. The power imbalance between nurses and medical healthcare professionals was related to hierarchical structures favouring the latter by McAndrew *et al.* (2018), who emphasized that medical values predominate over nursing values in the organizations in which they work, inhibiting nurses from executing their holistic role, and impeding the development of advanced nursing practice and nursing advocacy for patients in care decisions. The decision-making hierarchy often encourages physicians to ignore and disregard nurses’ opinions regarding patient care, contributing to the nurses’ moral distress ( Morley *et al.*, 2022; Shoorideh *et al.,* 2012).

From the results of this review, it was found that there was a strong hierarchical relationship between healthcare providers in the facilities where the studies were conducted. ICU nurses who are morally challenged felt powerless and helpless to stop treatment that they believed was ethically wrong, or where the care delivered did not meet required standards ( McAndrew *et al.,* 2018; Morley *et al.*, 2022; Prompahakul *et al.,* 2021). On this basis, we can emphasize the importance of a culture that promotes nurse autonomy and collaboration in decision-making related to patient care in different healthcare settings. Nurses perceived themselves as having little authority to make treatment decisions within the healthcare team. In many situations, nurses knew what was best for patients, but were unable to act due to a lack of practice and independence ( Prompahakul *et al.,* 2021). Concerns have been raised about the negative relationship between moral distress and autonomy and collaboration. Poor communication and collaboration can have serious consequences for patients and families if nurses do not feel valued in their professional interactions ( McAndrew *et al.,* 2018).

Inadequate resources, such as medical supplies, accessible beds, and staff, were another source of moral distress at the organizational level, as they undermined nurses’ ability to provide the best possible care to patients ( Henrich *et al.,* 2016; Prompahakul *et al.,* 2021). Specific safety risks were reported, such as one case in which management pressed nurses to care for three patients at once, while also providing break relief for a fourth patient, which the nurses did not believe was safe ( Henrich *et al.,* 2016).

Nurse empowerment and organizational support appear to be important factors in reducing moral distress among ICU nurses ( Altaker *et al.,* 2018; Browning, 2013; Henrich *et al.,* 2016; McAndrew *et al.,* 2018; Morley *et al.*, 2022; Prompahakul *et al.,* 2021; Shoorideh *et al.,* 2012). A study conducted to examine the relationship between moral distress and psychological empowerment among critical care nurses found that the total psychological empowerment score was negatively correlated with the frequency of moral distress, and multiple regression analysis revealed that empowerment was a significant predictor of the frequency of moral distress ( Browning, 2013).

Empowerment of the individual nurse has been the focus of numerous educational interventions, particularly in the area of end-of-life care and team building, but has not resulted in a reduction in moral distress levels ( Altaker *et al.,* 2018), implying that more knowledge and perceptions of empowerment are not sufficient to reduce the moral burden of ICU nurses; actual implementation of nurse empowerment in the organization is required to reduce ICU nurse moral distress, although a roadmap to facilitate this remains lacking.

The powerlessness of ICU nurses described above was not limited to their interactions with other healthcare professionals and organizational administrators but extended to their exchanges with service users. Several studies indicated that inappropriate and unnecessary treatments directed by patients’ family members were among the main causes of moral distress among nurses ( Browning, 2013; Henrich *et al.,* 2016; McAndrew *et al.,* 2018; Prompahakul *et al.,* 2021). Surrogates are influential decision makers in some countries when patients lack decision-making capacity, which is relatively common in ICU, and healthcare professionals can be subject to legal action if they do not adhere to surrogates’ wishes, even if they perceive these to be contrary to the interests of the patients themselves. As a result, some participants expressed moral concerns when they knew that the decision chosen was inappropriate for a patient, but they had no choice but to follow the surrogate’s decision because of the latter’s power within the care decision-making process ( Prompahakul *et al.,* 2021).

This may be related to prosaic issues related to the amenities offered to service users by critical care facilities. Families often have an interest in maximizing patients’ stay and treatment in ICU, where healthcare professionals undertake all care activities (including for activities of daily living), and which are usually under the umbrella of ICU medical costs covered by insurance schemes, thus families may ignore options to transfer patients to other units or even to discharge them from hospital when the nurse may consider this to be the best option for the patient.

In other studies, it was found that some patients’ family members refused treatment for financial reasons, or because of the burden of caring for a patient, even if the patient had a high chance of recovery. In such cases, physicians often agreed to discontinue treatment without first attempting to convince the patients’ families, which angered nurses because they had no control over the situation ( Choe *et al.,* 2015; Prompahakul *et al.,* 2021).

### End-of-life care

The reviewed studies concurred that moral distress was common among ICU nurses during the end-of-life decision-making process ( Browning, 2013; Forozeiya *et al.,* 2019; Henrich *et al.,* 2016; Prompahakul *et al.,* 2021; Sannino *et al.,* 2015, 2019). Almost 80% of the morally distressing situations mentioned by participants in Prompahakul *et al.,*'s (2021) study were end-of-life situations, including unnecessary medical treatments, futile actions, and giving false hope to patients and families at the end of life. According to Henrich *et al.* (2016), nurses were concerned when aggressive care continued, even when the decision to switch to comfort care was made, when the decision was made on a weekend or at night; physicians commonly wanted to wait until after the weekend or until the next morning to implement the plan, for their own convenience rather than patients’ best interests. This finding was confirmed by Prompahakul *et al.* (2021), who found that ICU nurses were disturbed when they saw patients suffering as a result of overly aggressive and unnecessary treatments that they believed were not in the best interest of the patient

Futile care has been described in several studies as a factor that triggers moral distress among ICU nurses ( Andersson *et al.,* 2022; Asayesh *et al.,* 2018; Hiler *et al.,* 2018; Karagozoglu *et al.*, 2017; Prompahakul *et al.,* 2021; Shoorideh *et al.,* 2012). Futile care can be defined on the basis of survival or subsequent quality of life, and includes aggressive treatments or end-of-life interventions in patients with very low life expectancy or chance of recovery ( Asayesh *et al.,* 2018). According to Shoorideh *et al.* (2012), ICU nurses suffer moral distress due to unnecessary testing, unnecessary and expensive medications for patients nearing the end of life, unnecessary counselling, and cardiopulmonary resuscitation ( Shoorideh *et al.,* 2012). In critical care, the failure to set limits on futile treatments may exacerbate the experience of moral suffering in nurses ( McAndrew *et al.,* 2018)

Deceptive information and false hope for patients and relatives were cited in several studies as a distressing act that caused moral distress for ICU nurses ( Andersson *et al.,* 2022; Browning, 2013; Forozeiya *et al.,* 2019; Morley *et al.*, 2022; Prompahakul *et al.,* 2021). Nurses’ distress over withholding information from patients’ families was compounded by a stark discrepancy between what they saw physicians say directly to families and what they then said in private ( Forozeiya *et al.,* 2019), affirming a point highlighted by Choe *et al.,* (2015). This information-framing resulted in families not receiving a complete picture of their patients’ deteriorating condition, leading to prolonged and aggressive care for dying patients. End-of-life care planning, including how physicians plan for end-of-life care and the extent of family involvement in end-of-life care decisions, was another aspect identified in two studies as contributing to moral distress ( Forozeiya *et al.,* 2019; Henrich *et al.,* 2016).

### Ineffective team function

Related to the hierarchical powerlessness described previously, poor communication and lack of cooperation with other healthcare professionals (particularly physicians) in caring for critically ill patients was a major cause of moral distress among ICU nurses in the articles reviewed ( Choe *et al.,* 2015; Henrich *et al.,* 2016; Karagozoglu *et al.*, 2017; Karanikola *et al.,* 2014; McAndrew *et al.,* 2018; Prompahakul *et al.,* 2021; Shoorideh *et al.,* 2012). When nurses reported higher levels of moral distress, they cited communication with the physician as a cause of medication errors ( McAndrew *et al.,* 2018). Similarly, Henrich *et al.* (2016) emphasized that ineffective physician communication with families was identified by nurses and other health professionals as a source of distress.

Distress from not being heard during inter-professional conversations about end-of-life care typically magnified ICU nurses’ sense of powerlessness, despair, and frustration ( McAndrew *et al.,* 2018). Many studies highlighted many inappropriate peer behaviours that increased feelings of moral distress ( McAndrew *et al.,* 2018; Morley *et al.*, 2022; Prompahakul *et al.,* 2021). Neglecting patients and treating them unequally due to social hierarchy are unethical and morally questionable behaviours that triggered moral distress when encountered ( Prompahakul *et al.,* 2021).

Working with incompetent healthcare providers was another problem addressed in several studies ( Andersson *et al.,* 2022; Browning, 2013; Henrich *et al.,* 2016; Karagozoglu *et al.*, 2017; McAndrew *et al.,* 2018; Prompahakul *et al.,* 2021; Sannino *et al.,* 2019). The nurses in the study conducted by Prompahakul *et al.,* (2021) noted that their colleague’s incompetence threatened the patient’s integrity due to delayed treatment or inappropriate pain management. In another study, supporting a physician who provides incompetent care was found to be one of the main causes of the frequency and intensity of moral distress experienced by ICU nurses ( McAndrew *et al.,* 2018).

In two studies, conflicting care plans of the attending physicians for critically ill patients were cited as a cause of moral distress ( Henrich *et al.,* 2016; Shoorideh *et al.,* 2012). Henrich *et al.* (2016) highlighted that the constant change in the care plan each week as new attending physicians took responsibility for patients was very stressful, as nurses had to explain to families why the direction of care had changed, especially if they disagreed with the change.

According to recent data, formal communication processes that involve the entire team in departmental ethics discussions and nursing rounds, or that provide ethics or morale-boosting counselling services, can reduce factors contributing to moral distress ( Altaker *et al.,* 2018, p. 300).

### Personal characteristics

Based on the findings of the current review, the personal characteristics of nurses may play an important role in moral distress. Hiler *et al.* (2018) concluded that the age of ICU nurses was a statistically significant predictor of the severity of moral distress experienced (i.e., age was negatively correlated with moral distress). Similar findings were highlighted by Sannino *et al.* (2019), as they found a negative moderate correlation between ICU nurses’ age and level of moral distress. However, Browning (2013) reached the opposite conclusion, finding a positive relationship between the age of ICU nurses and their moral distress, emphasizing that the intensity of moral distress increased with the age of the nurse.

This discrepancy in findings suggests that the relationship between age and moral distress is complex and may be influenced by various contextual factors. For example, younger nurses may be more adaptable to the evolving demands and stressors of the ICU environment, whereas older nurses, despite their experience, might feel a deeper sense of responsibility and ethical commitment, leading to higher moral distress. Additionally, institutional support and the nature of the ICU team dynamics might play a role in how age impacts moral distress. It could be beneficial for future research to explore the mediating factors such as coping mechanisms, support systems, and institutional policies that might influence this relationship.

Nurses with a higher educational level were found to experience higher intensity of moral distress than those with relatively less education in a study by Altaker *et al.* (2018, p. 299), while Shoorideh and Ashktorab (2015) found no significant correlation.

This finding may indicate that more educated nurses have heightened ethical awareness and are perhaps more critical of clinical practices that do not align with their professional standards. Their advanced education may equip them with a deeper understanding of the ethical implications of their actions, making them more susceptible to moral distress when they perceive a conflict between their knowledge and the care being provided. Conversely, less educated nurses might not perceive these conflicts as intensely, or they might lack the confidence to challenge the status quo.

A negative correlation was found between duration of caregiving experience and level of moral distress by Patrizio Sannino *et al.* (2019, p. 5), contrary to the results of other studies which reported a positive correlation between work experience and the level of moral distress ( Asayesh *et al.,* 2018; Shoorideh & Ashktorab, 2015). This inconsistency could be attributed to varying definitions of "experience" across studies or the different environments in which the studies were conducted. It is plausible that nurses with longer tenure in the ICU develop better coping strategies and resilience against moral distress, whereas those with less experience might struggle more with the emotional demands of the job. Alternatively, more experienced nurses might feel greater moral distress due to a cumulative effect of exposure to ethically challenging situations over time.

McAndrew *et al.* (2018) found that female nurses reported more moral distress than their male counterparts, while Shoorideh and Ashktorab (2015) found no significant correlation between gender and moral distress. Conversely, Sannino *et al.* (2015) reported that male nurses experienced a higher level of moral distress in a significantly higher percentage of cases than female nurses. These gender-related findings highlight the need to consider sociocultural and psychological factors that might influence how male and female nurses experience and report moral distress. Gender-specific socialization patterns and expectations could shape how nurses perceive and cope with morally distressing situations. For instance, female nurses might be more emotionally expressive and thus more likely to report higher levels of distress, whereas male nurses might underreport their distress due to societal expectations of emotional stoicism.

Overall, these findings underscore the importance of considering individual nurse characteristics when addressing moral distress. Healthcare institutions should tailor their support programs to address the specific needs of nurses based on their age, education level, experience, and gender. Further research should also aim to disentangle the complex interplay of these factors to develop more effective interventions to mitigate moral distress among ICU nurses.

### Limitations

The literature included comprised full-text and research studies, so the content available in philosophical treatises, editorials, and dissertations could have extended the findings reported in this article.

Because the focus of the study was mainly on critical care settings, any generalizations of the findings are applicable only to this area of practice. However, by including neonatal and paediatric critical care nurses’ experiences in this study, the findings could be generalized to a broader range of critical care nurses.

Additionally, the study only included documents in English. Considering the global impact of the COVID-19 pandemic, which has significantly influenced the moral distress of intensive care nurses worldwide, many studies on this topic have been published in languages other than English, and their exclusion may have limited the comprehensiveness of our review. Furthermore, we did not include searches in certain relevant biomedical databases, such as PsycINFO, nor did we incorporate gray literature sources. Including these additional sources could have provided a more extensive and nuanced understanding of the factors contributing to moral distress among ICU nurses.

## Conclusion

This review has highlighted the factors that contribute to moral distress in critical care nurses. Many of the associated factors are due to the organizational climate and the nature of the ICU clinical environment. It is suggested that further studies should be conducted to examine the effects of some variables on moral distress, such as nurse autonomy, job satisfaction, length of duty hours, and nurses’ personal characteristics, which might exacerbate or cause moral distress.

Therefore, descriptive and intervention studies, including both experimental and action research, are needed to investigate the causal relationship between the identified variables, and to determine the best strategy to help ICU nurses cope with and reduce the intensity of moral distress. Comparative studies are required to better understand the actual factors associated with moral distress, such as comparing the intensity of moral distress and contributing factors with a Magnet hospital, to examine the impact of organizational factors designed to improve nursing satisfaction and retention on moral distress.

## Data Availability

Figshare: Factors Contributing to Moral Distress Among Intensive Care Nurses: A Scoping Review. https://doi.org/10.6084/m9.figshare.21422514.v1. ( Ahmad *et al., *2022). The project contains the following underlying data:
• File 1- PubMed search strategy performed July 13, 2022.jpg. (Search strategy used in this study). •
Figure 1-PRISMA 2020 flow diagram.jpg. (Flowchart of study). •
Table 1 – Eligibility criteria.jpg. (Inclusion criteria for studies). •
Table 2 – Summary of included articles.docx. (List of final 17 articles in results). •
Table 3 – General characteristics of included studies.jpg. (Characteristics of 17 final studies). •
Table 4 – Content analysis.jpg. (Content analysis of final 17 studies). Figshare: Factors Contributing to Moral Distress Among Intensive Care Nurses: A Scoping Review. https://doi.org/10.6084/m9.figshare.21422514.v1. ( Ahmad *et al., *2022). This project contains the following extended data:
• Blank example of data extraction form.xlsx (blank extraction form used in this study). Zenodo: PRISMA-Scr checklist for ‘Factors Contributing to Moral Distress Among Intensive Care Nurses: A Scoping Review’. https://doi.org/10.6084/m9.figshare.21422514.v1. ( Ahmad *et al., *2022). Data are available under the terms of the Creative Commons Zero “No rights reserved” data waiver (CC0 1.0 Public domain dedication).
