# Supplementary material for: Factors contributing to moral distress among intensive care nurses: A scoping review
Source: F1000Res. 2024 Jul 15;11:1574. Originally published 2022 Dec 23. [Version 2] doi: 10.12688/f1000research.127120.2 (PMC11249504; doi:10.12688/f1000research.127120.2)
Supplement: Peer review history

# Reviewer report: Reviewer response for version 2

**DOI:** 10.5256/f1000research.168992.r303155

**Published:** 2024-07-17

**Authors:** Ratchaneekorn Upasen

**Affiliations:** 1 Faculty of Nursing, Chulalongkorn University, Bangkok, Thailand

1. Method section mentioned that relevant studies published between 2011 to 2022. so, in the text "method section" should be the same period.

Are the rationale for, and objectives of, the Systematic Review clearly stated?

Yes

Is the statistical analysis and its interpretation appropriate?

Yes

If this is a Living Systematic Review, is the ‘living’ method appropriate and is the search schedule clearly defined and justified? (‘Living Systematic Review’ or a variation of this term should be included in the title.)

Yes

Are sufficient details of the methods and analysis provided to allow replication by others?

Yes

Are the conclusions drawn adequately supported by the results presented in the review?

Partly

Reviewer Expertise:

My area is mental health and psychiatric nursing

I confirm that I have read this submission and believe that I have an appropriate level of expertise to confirm that it is of an acceptable scientific standard.

# Reviewer report: Reviewer response for version 2

**DOI:** 10.5256/f1000research.168992.r303156

**Published:** 2024-07-17

**Authors:** Luca Giuseppe Re

**Affiliations:** 1 ASST Grande Ospedale Metropolitano Niguarda, Milan, Italy

Dear Editor, dear authors, after carefully reading the second version of your article I verified that you have applied my suggestions. So as far as I'm concerned I give full approval to the article.

Are the rationale for, and objectives of, the Systematic Review clearly stated?

Yes

Is the statistical analysis and its interpretation appropriate?

Yes

If this is a Living Systematic Review, is the ‘living’ method appropriate and is the search schedule clearly defined and justified? (‘Living Systematic Review’ or a variation of this term should be included in the title.)

Not applicable

Are sufficient details of the methods and analysis provided to allow replication by others?

Partly

Are the conclusions drawn adequately supported by the results presented in the review?

Partly

Reviewer Expertise:

Clinical Nursing Pediatric Nursing Systematic Review Meta-analysis

I confirm that I have read this submission and believe that I have an appropriate level of expertise to confirm that it is of an acceptable scientific standard.

# Reviewer report: Reviewer response for version 1

**DOI:** 10.5256/f1000research.139595.r295684

**Published:** 2024-07-15

**Authors:** Ratchaneekorn Upasen

**Affiliations:** 1 Faculty of Nursing, Chulalongkorn University, Bangkok, Thailand

Thank you very much for giving me to comments and suggests this manuscript. There are some comments, please see as below:

 1. In the abstract mentions relevant studies published between 2011 to 2022, but the text states that ..articles published in the period between January 2010 to July 2022... please check again

 2. Study selection part, please provide key words to search each data based

 3. Data analysis section, please explain more how to analyze the qualitative data? which was method used in this study?

 4. The qualitative studies just present theme or categories about related factors to moral distress in ICU nurses, so the data was not tested that associated or related to the moral distress. How to make sure that they are related factors or associated the moral distress.

 5. Sub theme "Nurse autonomy" How to related to the major theme "powerlessness" Please explain in discussion section.

Are the rationale for, and objectives of, the Systematic Review clearly stated?

Yes

Is the statistical analysis and its interpretation appropriate?

Yes

If this is a Living Systematic Review, is the ‘living’ method appropriate and is the search schedule clearly defined and justified? (‘Living Systematic Review’ or a variation of this term should be included in the title.)

Yes

Are sufficient details of the methods and analysis provided to allow replication by others?

Yes

Are the conclusions drawn adequately supported by the results presented in the review?

Partly

Reviewer Expertise:

My area is mental health and psychiatric nursing

I confirm that I have read this submission and believe that I have an appropriate level of expertise to confirm that it is of an acceptable scientific standard, however I have significant reservations, as outlined above.

# Reviewer report: Reviewer response for version 1

**DOI:** 10.5256/f1000research.139595.r295677

**Published:** 2024-07-08

**Authors:** Luca Giuseppe Re

**Affiliations:** 1 ASST Grande Ospedale Metropolitano Niguarda, Milan, Italy

I thank the Editor for the opportunity to review this very interesting study, which concerns a topic very dear to me that has returned to the fore during the period of the COVID-19 pandemic.

 In my opinion, however, for indexing of the manuscript to be possible, it is necessary to overcome some methodological limitations that emerged after careful reading.

 I've included my notes below.

 Abstract

 Methods Section

 I would suggest the authors move the part starting with “After Removal 63 Duplicates (…)” and ending with “so 17 Studies (…)” to the beginning of the Results section

 Main text

 Methods Section

 Subsection Research Methods

 I would kindly ask the authors to report the day, month and year in which (or starting from which) they carried out the query of the biomedical databases

 Subsection Study selection

 Also in this case, I would suggest the authors to move the part that begins with “The initial search produced a total of 1,005 articles (…) and ends with “(…) therefore 17 studies were finally analyzed (see Figure 1)” to beginning of the Results section, as well as Figure 1.

 I would suggest the authors move Table 2 to the Results section to the subsection “General characteristics of the included studies”

 Discussion section

 Subsection Personal characteristics

 The authors illustrate the contents that emerged from the documents they found, but they should also express their own considerations, a typical characteristic of the Discussion section

 Subsection Limitations

 I would suggest the authors highlight two other important limitations: a) they only included documents in English (this limitation is important, in reference to the fact that, for example, the COVID-19 pandemic has had a great impact on moral distress of intensive care nurses around the world, hence the flourishing of many studies in languages ​​other than English); b) they did not query a biomedical database relevant to the topic, such as PsycINFO, and did not consult gray literature resources

Are the rationale for, and objectives of, the Systematic Review clearly stated?

Yes

Is the statistical analysis and its interpretation appropriate?

Yes

If this is a Living Systematic Review, is the ‘living’ method appropriate and is the search schedule clearly defined and justified? (‘Living Systematic Review’ or a variation of this term should be included in the title.)

Not applicable

Are sufficient details of the methods and analysis provided to allow replication by others?

Partly

Are the conclusions drawn adequately supported by the results presented in the review?

Partly

Reviewer Expertise:

Clinical Nursing Pediatric Nursing Systematic Review Meta-analysis

I confirm that I have read this submission and believe that I have an appropriate level of expertise to confirm that it is of an acceptable scientific standard, however I have significant reservations, as outlined above.

# Response

**Published:** 2024-07-10

**Authors:** Amina Mussa Ahmad

**Affiliations:** University of Sharjah, United Arab Emirates

Dear Dr. Luca,

 Thank you for the opportunity to review our study and for your valuable comments. Your feedback has significantly enhanced the readability and quality of our article. We deeply appreciate your thorough review and insightful suggestions.

 We are pleased to inform you that almost all points raised in your review have been addressed and modifications have been made accordingly in the revised version of the study. Below is a summary of the changes made:

**Note 1: Abstract-Methods Section**

- The suggested part has been moved to the Results section.

**Note 2: Methods Section-Subsection Research Methods**

- The month and year from which the search was initiated have been added to the Methods section, specifically highlighting that the search started in July 2022.

**Note 3: Subsection Study Selection**

- The specified part has been moved to the beginning of the Results section as suggested. As for Table 2, we have chosen to keep it in the Data Analysis section as it is intended to be the data extraction table that summarizes the findings of the results, which are illustrated in Table 3.

**Note 4: Discussion Section-Subsection Personal Characteristics**

- We have added a discussion of the findings along with the authors' assumptions and considerations, as you recommended. Thank you for this valuable note.

**Note 5: Subsection Limitations**

- We have considered your valid points and added the mentioned limitations to the Limitation section, acknowledging the exclusion of non-English documents and the lack of querying certain relevant biomedical databases and gray literature resources.

Once again, thank you for your constructive feedback and for helping us improve our study.

# Reviewer report: Reviewer response for version 1

**DOI:** 10.5256/f1000research.139595.r158631

**Published:** 2023-02-20

**Authors:** Intima Alrimawi

**Affiliations:** 1 School of Nursing, Georgetown University, Washington, DC, USA

This scoping review explores the factors that would trigger moral distress among intensive care nurses by utilizing the scoping review approach. This is a very important topic that was not covered in other scoping reviews. 

 In the methodology section, the researchers thoroughly discussed the scientific approach that they followed to conduct this review. 

 The result section needs to be developed further, further discussion is needed around the selected studies and the final themes that did emerge from them. 

 The discussion section needs to take the themes to the next level and discuss the potential implication of each point. For example, if you think ineffective team function was a main trigger for moral distress, then what is the implication of this in the current practice, and how can we overcome this on the ground? 

 The authors can also add a recommendation section.

 This scoping review explores the factors that would trigger moral distress among intensive care nurses through utilizing the scoping review approach and found that powerlessness, end-of-life care, ineffective teamwork, and the personal characteristics of ICU nurses were the main influencing factors of moral distress. The main strength of this article is that it discusses a main subject that would affect the nurses' practices by utilizing the systematic and scientific approach of scoping review. On the other hand, the weakness of this paper would be the lack of critical thinking around the meaning of this finding and how this can change in the future.  

 The rationale, and objectives of, the Systematic Review were clearly stated. The detailed process of the scoping review was clearly described, and the conclusion was adequately supported by the results presented in the review.

Are the rationale for, and objectives of, the Systematic Review clearly stated?

Yes

Is the statistical analysis and its interpretation appropriate?

Not applicable

Are sufficient details of the methods and analysis provided to allow replication by others?

Yes

Are the conclusions drawn adequately supported by the results presented in the review?

Yes

Reviewer Expertise:

My research focuses on improving the quality of care for vulnerable families and children with complex health concerns or critical illness and developing health services mainly in low and middle-income countries, as well as developing the quality of nursing education.

I confirm that I have read this submission and believe that I have an appropriate level of expertise to confirm that it is of an acceptable scientific standard.
